# Self‐Assembled Rhodium Nanoantennas for Single‐Protein UV SERS

**DOI:** 10.1002/nap2.70137

**Published:** 2026-05-23

**Authors:** Yanqiu Zou, Nicco Corduri, Francesco D'Amico, Karol Kołątaj, Huaizhou Jin, Zhenrong Zheng, Yifan Yu, Jie Liu, Shukun Weng, Ali Douaki, Roman Krahne, Jerome Wenger, Shangzhong Jin, Guillermo Acuna, Denis Garoli

**Affiliations:** ^1^ State Key Laboratory of Modern Optical Instrumentation, College of Optical Science and Engineering Zhejiang University Hangzhou China; ^2^ Department of Physics University of Fribourg Fribourg Switzerland; ^3^ Elettra Sincrotrone Trieste S.C.p.A. Basovizza Trieste Italy; ^4^ Key Laboratory of Quantum Precision Measurement, College of Physics Zhejiang University of Technology Hangzhou China; ^5^ Department of Chemistry Duke University Durham North Carolina USA; ^6^ Istituto Italiano di Tecnologia Genova Italy; ^7^ Dipartimento di Scienze e Metodi dell’Ingegneria Università degli Studi di Modena e Reggio Emilia Reggio Emilia Italy; ^8^ Aix Marseille Université, CNRS, Centrale Med, Institut Fresnel, AMUTech Marseille France; ^9^ College of Optical and Electronic Technology China Jiliang University Hangzhou China

## Abstract

Surface‐enhanced Raman scattering (SERS) provides critical insights into analyte structure, dynamic processes, and intermolecular interactions at the single‐molecule level. By exploiting the hotspot formed in the vicinity of plasmonic structures, SERS has become an established tool for fundamental biological research, particularly for early‐stage disease diagnostics. DNA Origami, with its high addressability, enables both the assembly of plasmonic nanostructures with nanometric accuracy, and the deterministic placement of a single analyte molecule precisely at the hotspot. Most DNA Origami based nanoantennas rely on gold or silver nanoparticles (NPs), whose plasmonic resonances are confined to the visible spectrum, hindering the exploitation of the strong biomolecules' absorbance in the UV range. To benefit from the supplementary Raman signal enhancement in the UV, we extended the operational range of SERS with a deterministic strategy for self‐assembling (UV)‐plasmonic dimer antennas using rhodium nanocubes. Herein, we leverage this tailored architecture to systematically investigate its performance for single‐molecule UV‐SERS and demonstrate how biofabricated Rh‐dimers can be used to detect the characteristic SERS signal of a single streptavidin molecule linked at the dimer's gap. Our results are validated by polarization‐dependent measurements on a single dimer, which show the expected modulation of the SERS signal with dimer orientation only for DNA origami structures hosting a protein in the hotspot. This work establishes a highly sensitive and polarization‐tunable UV‐SERS platform and lays a solid foundation for label‐free optical investigation and bio‐spectroscopy of individual biomolecules in the UV spectral range.

## Graphical abstract

The use of biofabrication (DNA origami) to prepare Rh nanocubes dimer enables the realization of a plasmonic platform for single‐molecule UV‐SERS. A reproducible, self‐assembly technique for nanofabrication, combined with UV spectroscopy extended the application of single molecule SERS to the UV spectral range.

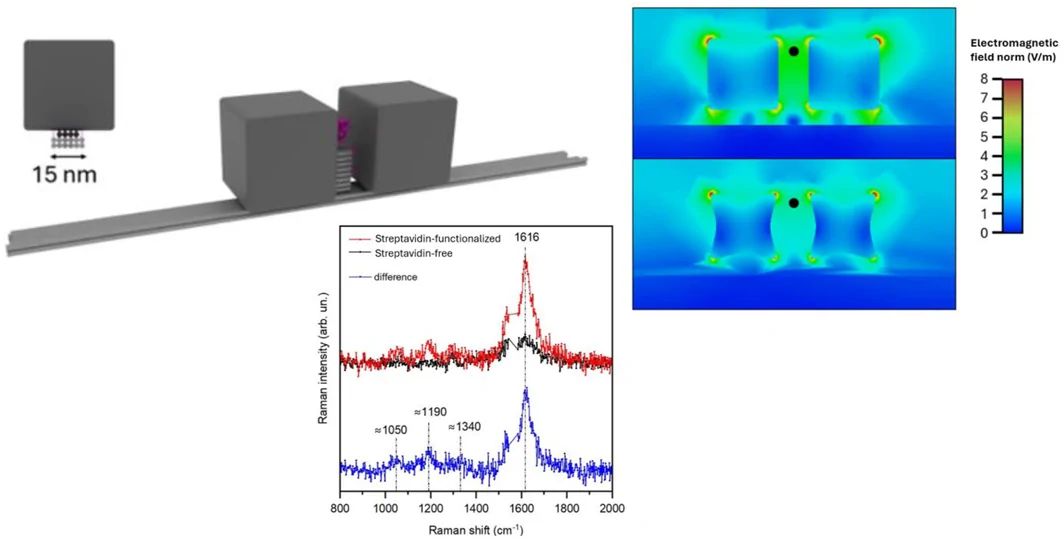

## 1 Introduction

Surface‐enhanced Raman scattering (SERS) is a nondestructive, label‐free and highly sensitive technique that provides molecular fingerprinting, enabling deep understanding of biomolecular structure and dynamics [1, 2, 3, 4, 5, 6, 7]. This technique relies critically on nanoscale plasmonic hotspots that generate enhanced localized electromagnetic fields, thereby enabling the use of Raman spectroscopy for single‐molecule detection [8, 9, 10]. Diverse SERS platforms have been reported [11], with examples based on colloidal nanoparticles (NPs) [12], nanoporous systems [13], and nanostructures or nanoantennas prepared via different lithographic methods [14, 15, 16]. In general, conventional bottom‐up methods often lacks nanometric precision and reproducibility to assemble hotspots with consistent and enhanced optical properties even for homogeneous substrates, whereas top‐down fabrication methods are usually expensive, and have difficulties reaching very high enhancement factors [13, 17, 18]. Furthermore, with these techniques it is extremely challenging to deterministically position an individual analyte within the hotspot.

To overcome these limitations, biofabricated DNA origami [3, 19, 20] has emerged as a powerful bottom‐up synthesis strategy by assembling functionalized nanoparticles with synthetic oligonucleotides into nanometric plasmonic structures [21, 22, 23, 24]. The same addressable DNA scaffold can also host a single target molecule directly within the plasmonic hot spot [25], fulfilling both requirements for reliable single‐molecule SERS. For instance, Kosti Tapio et al. reported a method for utilizing DNA origami to precisely position single analyte molecules into the plasmonic hotspot, thereby enabling single‐molecule SERS measurements of various dyes and proteins [3]. In a similar approach, Schuknecht et al. reported DNA origami‐based assembly of tip‐to‐tip gold nanorod dimers with controlled gaps of approximately 8 nm, enabling label‐free single‐protein SERS by specifically capturing target proteins within the plasmonic hotspot [26]. So far, the practical implementation of such DNA‐assembled nanoantennas has been limited to the visible/near‐infrared (NIR) spectral range, as gold or silver NPs are predominantly employed with limited extensions into high‐index electric materials [22, 24, 26, 27, 28]. However, this spectral range is severely limiting SERS applications for two reasons. First, fluorescent background in the visible range is typically significant for plasmonic platforms; second, most biomolecules have small intrinsic Raman cross‐sections in the visible and NIR regions [29]. Therefore, higher excitation energy (UV radiation) can be used to reduce the background fluorescence and can lead to UV resonance Raman enhancement thanks to the intrinsic absorbance of the biomolecules in this spectral range [30, 31]. Nanostructures and NPs based on metals such as aluminum (Al), magnesium (Mg), rhodium (Rh) and gallium (Ga) can exhibit localized surface plasmon resonances (LSPRs) in the UV to deep‐UV wavelengths [29, 32, 33, 34, 35, 36]. Recent works have reported the integration of two or more plasmonic materials in the same system introducing additional tunability in the spectroscopic properties [37, 38]. For instance, in a recent research we investigated UV‐SERS and plasmon‐driven photochemistry/photodegradation using a composite platform of nanoporous Al decorated with Rh NPs [39]. Although this approach provided valuable insights from averaged UV‐SERS spectra, it offered limited control over the morphology, size, and spatial distribution of the Rh NPs. More recently, we developed a strategy for constructing well‐defined, programmable Rh nanocube (NC) dimers on DNA origami scaffolds, which can precisely host one biomolecule at the hotspot. This system was then used to demonstrate single‐protein detection by means of UV fluorescence [40]. In fact, Rh NCs are ideal UV‐plasmonic structures due to their strong LSPR in the UV region and excellent aqueous stability, properties that have been directly leveraged for effective UV‐SERS [41, 42]. Building upon this tailored UV‐plasmonic platform, here we demonstrate how it can be used to achieve single‐protein UV‐SERS detection. Furthermore, we show a pronounced synergistic enhancement effect between the aluminum film substrate and the Rh‐NCs‐dimer nanoantenna by using well‐designed micropattern, showing that the UV‐SERS spectrum of functionalized streptavidin inside the hotspot can be detected consistently only on top of an aluminum film. Our observations are further confirmed by polarization resolved measurements, confirming numerical simulations [43, 44, 45, 46, 47], thus verifying that the SERS signal of streptavidin is maximized when the polarization of the incident laser on the sample is aligned parallel to the main axis of the single NCs dimer excited within the laser spot. Overall, this study expands the applicability of programmable SERS and DNA origami devices down to the UV range broadening the capabilities to interrogate single label‐free proteins.

## 2 Results

### 2.1 Concept and Design

Figure 1a depicts the strategy to fabricate Rh NC dimers. In brief, a rectangular DNA origami with dimensions of 180 × 5 × 15 nm (length × height × width) and an 8.5 nm wide “mast” at the center is modified to incorporate through DNA hybridization two Rh NCs. At the dimer hotspot, which coincides with the upper part of the mast, a biotin modification enables the incorporation of a single streptavidin protein. This approach enables the fabrication of UV antennas based on Rh NC dimers with and without a single protein at the hotspot for control measurements [40]. Figure 1b includes a TEM image of the dimer structures, whereas additional TEM micrographs are reported in (Supporting Information S1: Note #1). From TEM image analyses over a large set of dimers, an average interparticle distance of 10.7 ± 3.2 nm using the DNA origami technique has been obtained. Two sets of Rh NCs have been considered, with size of 19 and 24 nm, respectively. The UV/Vis absorbance measurements revealed a broad resonance centered between 300 and 320 nm in buffer conditions, respectively for 19 and 24 nm Rh NCs (see Supporting Information S1: Note #2).

**FIGURE 1:**
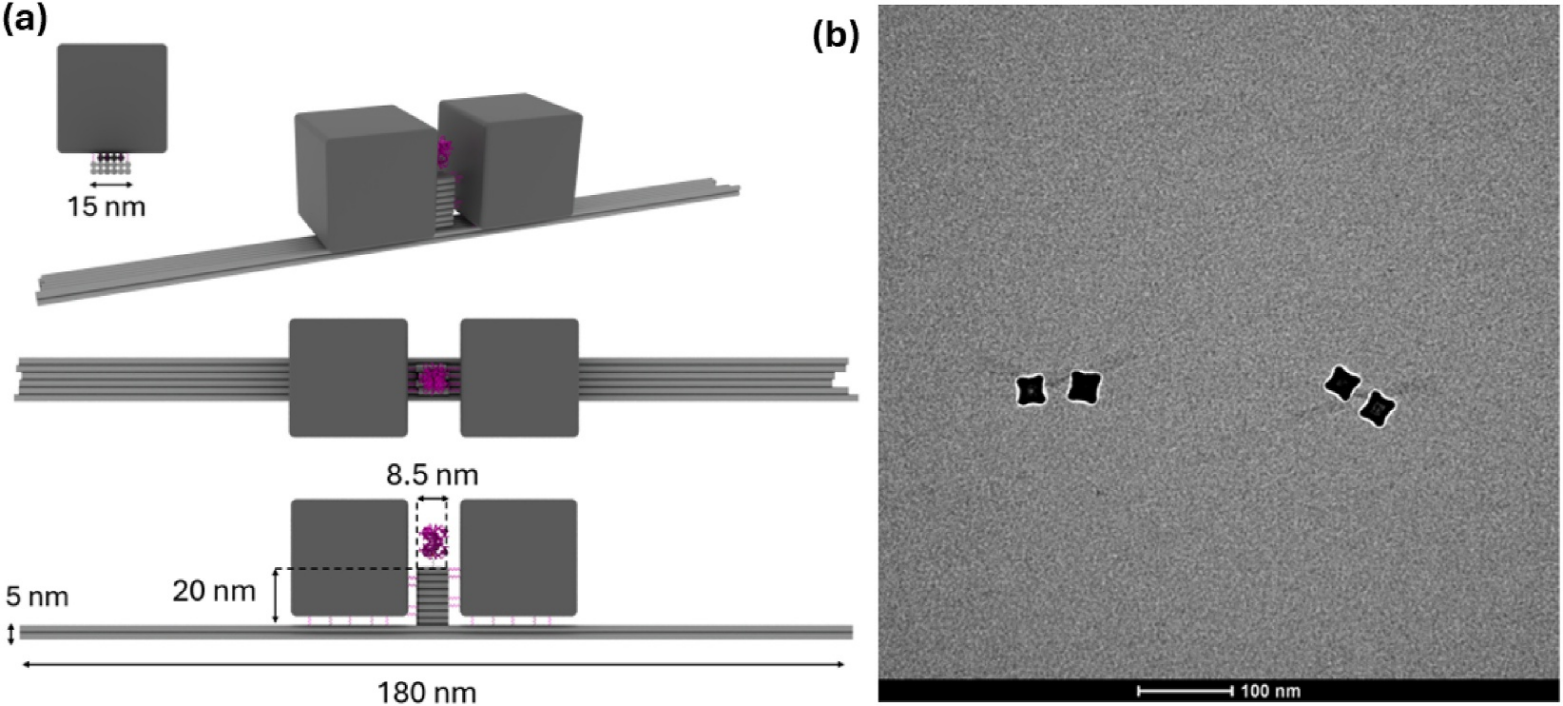
Sketch of the DNA origami employed for assembling an Rh NC dimer. (a) DNA functionalized Rh NCs are incorporated to DNA origami. The solution containing the Rh NC dimers was drop‐cast on top of porous aluminum films. (b) TEM image of DNA origami based 24 nm Rh NC dimers on top of an aluminum film.

SERS measurements were performed using two excitation wavelengths. First, to probe the response of Rh NC dimers near their absorbance maximum, a 325 nm laser was coupled to a HORIBA Raman system capable of acquiring spectroscopic 2D maps and equipped with an ultraviolet polarizer in the optical path, as schematically shown in Supporting Information S1: Figure S3 (Note #3). Second, for comparison with the most commonly used excitation wavelength in UV‐SERS, the nanostructures were investigated using a different setup (not capable of 2D mapping) coupled to a 266 nm laser and equipped with a half‐wave plate to rotate the incident polarization (see Supporting Information S1: Figure S4, Note #3). The use of this second excitation wavelength allows probing single molecules at an energy closer to the absorption bands of several aromatic amino acids, which exhibit characteristic absorption features at approximately 255 nm, 270 nm, and 286 nm, thereby enhancing the contribution of molecular UV absorbance [48]. It is worth noting that the morphology of the Rh NCs is not always perfectly cubic. In fact, they exhibit a slight concavity, which will be taken into account in the following discussion of the expected enhancement factor.

### 2.2 Validating Single‐Molecule and Specific DNA‐Protein Binding via UV‐SERS

For our single‐molecule UV SERS experiments, we selected streptavidin (molecular weight ≈ 60 kDa, diameter ≈ 5 nm) as a model protein [26, 49, 50]. Therefore, we added a biotin extension at the top of the DNA origami mast to precisely connect a single streptavidin molecule in the hotspot. This streptavidin–biotin linkage is exceptionally stable and specific, owing to a deeply buried binding pocket and cooperative conformational changes that yield ultra‐high affinity [51]. To validate the specificity and single‐molecule detection capability of our platform, we performed UV‐SERS mapping on the DNA‐origami‐based Rh NC dimers in the two configurations, without and with the presence of the single streptavidin protein at the hotspot, name “streptavidin‐free” and “streptavidin‐functionalized,” respectively. To note, although the Rh NCs dimers are prepared in solution, their deposition on the substrates used for the SERS analyses cannot be completely controlled. Therefore, the dimers can be positioned on the surface with the DNA origami laying on the surface (normal) or the dimer laying on the NPs (inverted) (see Supporting Information S1: Figure S5, Note #4—analyses on the effect of these two configurations on the electromagnetic field enhancement are reported in our recent publication Ref. [40]). Hence, it is reasonable to expect to detect SERS signals from the DNA origami from the samples prepared without the protein, wheras an overlap of DNA and protein vibration peaks are expected only in the case of protein detection.

Figure 2 reports the UV‐SERS results obtained from the 24 nm Rh NCs. We first collected comparative Raman mapping over a 70 × 70 μm^2^ area with a step size of 14 μm (see Figure 2a,c). The differences between the spectra collected on the samples with and without the protein are clearly observable. Considering the UV excitation energies, photodegradation effects cannot be completely avoided and they must be taken into consideration. In particular, we performed a detailed study on the UV‐induced photodegradation on a Rh‐Al plasmonic platform in recent works [36, 39]. In particular, exploring the effect of successive UV exposure on DNA and protein molecules, we were able to tune the exposure time and laser power to the values used here. Therefore, according to our previous experiments we selected exposure parameters compatible with reduced photodegradation (1800 gr/mm grating with 10% laser power and an integration time of 270 s per point).

**FIGURE 2:**
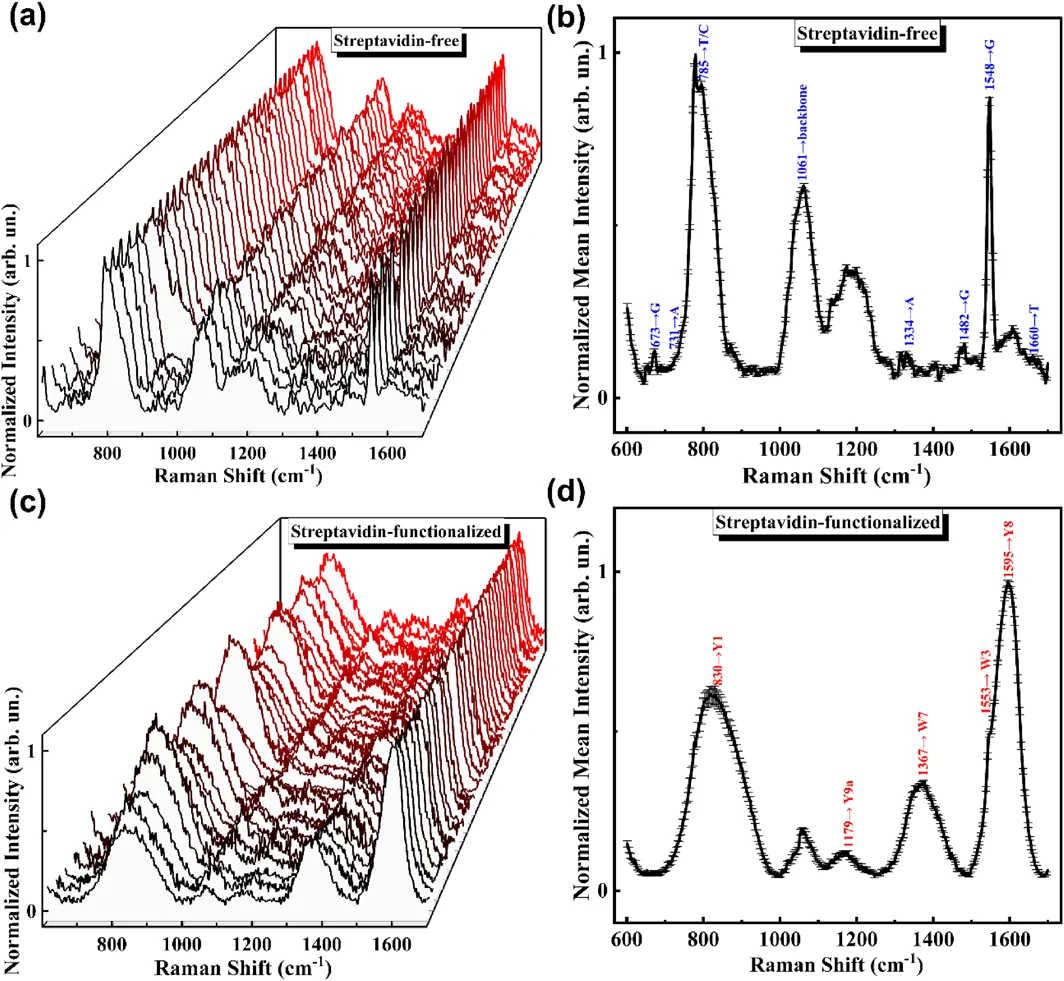
Validation of specific DNA‐protein binding and single‐molecule detection via UV‐SERS. (a) Normalized UV‐SERS mapping spectra obtained on aluminum‐based films functionalized without streptavidin (streptavidin‐free). Different lines represent different points on the sample. (b) Normalized averaged UV‐SERS spectrum with error bars calculated from the data in (a). Characteristic DNA peaks are labeled in blue: phosphate backbone (1061 cm^−1^), A (adenine, ∼731, ∼1334 cm^−1^), T (thymine, ∼785, 1660 cm^−1^), C (cytosine, ∼785 cm^−1^), and G (guanine, ∼673, ∼1482, and ∼1548 cm^−1^). (c) Normalized UV‐SERS mapping spectra obtained on aluminum‐based films functionalized with streptavidin captured inside the DNA Origami‐positioned Rh nanoantennas (streptavidin‐functionalized). Different lines also represent different points on the sample. (d) Normalized averaged UV‐SERS spectrum with error bars calculated from the data in (c). Observed peaks associated with streptavidin‐biotin are labeled: Y1 (∼830 cm^−1^), Y9a (∼1179 cm^−1^), W7 (∼1367 cm^−1^), W3 (∼1553 cm^−1^), and Y8 (∼1595 cm^−1^), corresponding to vibrational modes of tyrosine (Y) and tryptophan (W) residues.

The corresponding normalized average spectra are reported in Figure 2b,d. The normalized mean spectrum from streptavidin‐free sample clearly shows several peaks associated with the DNA molecules [52], as labeled in blue in Figure 2b. We also presented the averaged spectra with the difference spectrum in in Supporting Information S1: Figure S6c (Note #5). Notably, the spectral intensity remained stable within a 15% variation across the mapping area, which is most probably due to the probed Rh NCs dimers density, where we expect at least one nanostructure per laser spot for all tested points. In contrast, the averaged normalized spectrum obtained from the streptavidin‐functionalized sample exhibits pronounced additional broad Raman bands, which are assigned to streptavidin–biotin vibrational modes (Y1, Y9a, W7, W3 and Y8, as labeled in red in Figure 2d). The presence of these strong and broad protein bands, alongside the persistent and sharp DNA peaks at 1061 cm^−1^ (backbone) and 1548 cm^−1^ (guanine), calls for a detailed vibrational assignment to deconvolute the composite spectrum and confirm the origin of each feature. In this second case, the intensity of the Raman peaks varied significantly over the different points of the 2D map (Figure 2c and relative error bars in Figure 2d), especially in the averaged spectra in Supporting Information S1: Figure S6c (Note #5). This can be reasonably justified with the high sensitivity required to detect the single small protein in the nanogap between the Rh NCs. Moreover, for statistical reasons, we expect that not all the Rh NCs dimers linked the single protein in the gap.

To further visualize the spatial distribution of the DNA origami nanostructures, the Raman maps were integrated over the 1055–1067 cm^−1^ region, which corresponds to the characteristic DNA backbone peak at 1061 cm^−1^ (see Supporting Information S1: Figure S6a,b, Note #5). A detailed vibrational assignment for the range of our observed Raman bands is provided in Table 1. This analysis confirms that the broad feature bands in the protein‐bound spectra originate from characteristic modes of streptavidin. Specifically, according to the literature, peaks assignable to tryptophan residues (e.g., W7 at ∼1367 cm^−1^, W3 at 1553 cm^−1^) [26, 57, 58, 61] and tyrosine residues (e.g., Y1 at ∼830 cm^−1^, Y9a at ∼1179 cm^−1^, Y8 at ∼1595 cm^−1^) [26, 58, 59, 61] are clearly identified in our experimental normalized mean spectra in Figure 2c,d, which are absent in the control spectrum dominated by DNA signatures in Figure 2a,b. To quantitatively validate these assignments, the difference spectrum (streptavidin‐functionalized minus streptavidin‐free) in Supporting Information S1: Figure S6c (Note #5) was deconvoluted into individual Raman components, yielding an excellent fit (*R* ^2^ = 0.99606) with peaks corresponding to the vibrational modes of both the DNA backbone and streptavidin (see Supporting Information S1: Figure S6d, Note #5). Although the deconvoluted peak positions show slight deviations of a few wavenumbers from the literature values, these minor differences are well within the typical uncertainty range of UV‐SERS measurements and do not affect the reliability of the assignment. This clear spectroscopic distinction provides direct evidence for the specific capture of streptavidin at the ensemble level.

**TABLE 1:** SERS peak assignments for DNA‐origami scaffold and streptavidin.

Ours work (cm^−1^)	Assignment	Vibrational mode	References
DNA			
∼673	G	Ring breathing	[53, 54, 55]
∼731	A	Ring breathing	[27, 53, 54, 55, 56, 57]
∼785	T/C	Ring breathing	[53, 54, 55]
∼1061	Phosphate backbone	PO_2_^−^ symmetric stretch	[27, 54, 55, 57]
∼1334	A	Ring vibration (C–N stretch)	[53, 54, 55, 56, 57]
∼1482	G	Ring stretching/deformation coupling	[27, 53, 54]
∼1548	G	Purine ring stretching vibration	[53, 54]
∼1660	T	C=O/C=N stretch (DNA)	[53, 54]
Streptavidin‐biotin			
∼830	Y1	Tyrosine	[26, 58, 59, 60]
∼1179	Y9a	Tyrosine	[26, 58, 59, 61]
∼1367	W7	Tryptophan	[26, 58, 59, 61]
∼1553	W3	Tryptophan	[26, 58, 59]
∼1595	Y8	Tyrosine	[59]

Abbreviations: A, adenine; C, cytosine; G, guanine; T, thymine.

Further comparison of the UV‐SERS spectra between 24 and 19 nm Rh NCs is included in Supporting Information S1: Figure S7 (Note #6). In this case, the 2D mappings (225 points per sample) were obtained using low surface density samples, that is, we deposited the Rh NCs prepared on the origami starting from a diluted solution in order to obtain an inter‐dimers distance >> 1 μm). The maps are a first demonstration of the single molecule sensitivity and they show that the 24 nm dimers exhibit a higher density of bright hotspots and more intense spectral features associated with streptavidin (see Supporting Information S1: Figure S7). Statistical analysis of the peak intensities at ∼1595.7 cm^−1^ (see Supporting Information S1: Figure S8, Note #6) reveals that the 24 nm dimers produce ultra‐high‐intensity events (> 1000 a.u.), whereas the 19 nm sample shows no such signals. Based on these quantitative results, the 24 nm Rh NCs were selected for all subsequent experiments.

To proceed with the peak identification and discussion, it must be emphasized that in our previous study, we tested the UV‐SERS of BSA and L‐tyrosine (at high concentrations) using the same experimental system under 325 nm laser excitation [39]. The results showed that the spectra of proteins such as BSA and L‐tyrosine were characterized by broad, poorly resolved features spanning approximately 1500–1700 cm^−1^, with only a few discernible peaks (e.g., around 1606 cm^−1^). This spectral broadening is characteristic when the laser energy is not in strong resonance with the analytes' electronic transitions, leading to a low inherent Raman cross‐section and a signal dominated by a superposition of many weakly enhanced, unresolved vibrational modes, further convoluted by potential substrate‐molecule interactions [62]. In contrast, the streptavidin‐bound spectra presented herein, while also exhibiting broadened bands, show a set of distinct and reproducible features that enable a confident spectral deconvolution.

These results collectively demonstrate that our architecture not only generates a specific UV‐plasmonic response to protein binding but also achieves the spatial and spectral resolution necessary for detecting and spectroscopically identifying individual biomolecular interactions. To further validate the substrate specificity and spatial control of our platform, we also performed integrated UV‐SERS mapping on micropatterns comprising alternating stripes of aluminum and silicon (see Supporting Information S1: Note #7 and methods for details). The role of Al as substrate for the Rh‐NCs has been discussed in our recent work [40] where we showed how the “mirror” effect of the metallic substrate contributed to increase both the electromagnetic field confinement and enhancement and the collection efficiency.

Under low DNA‐origami assembly density (> 1 μm average inter‐dimer spacing), SERS signals from the DNA scaffold and the captured streptavidin were exclusively and reproducibly detected on the aluminum regions, whereas no streptavidin‐associated peaks were observed on the silica stripes (Supporting Information S1: Figure S9). This clear spatial contrast confirms that the observed UV‐SERS enhancement is intrinsically supported by the aluminum‐based plasmonic nanostructure and the enhancement provided by the Rh NCs (when deposited on Si) alone is not enough to ensure reproducible single molecule detection. Control experiments on Si substrates further support this conclusion: whereas a weak DNA‐related peak can occasionally be detected on silicon (Supporting Information S1: Figure S11, Note #9), the signal intensity and spectral richness are substantially lower than on aluminum. Moreover, control samples lacking Rh NCs showed no detectable SERS signals regardless of the presence of streptavidin (Supporting Information S1: Figure S12, Note #9), confirming that the observed enhancement is primarily mediated by the plasmonic activity of the Rh NC dimers. The normal Raman spectrum of bulk streptavidin (Supporting Information S1: Figure S13, Note #9) further confirms that the characteristic SERS peaks arise from plasmonic enhancement, as the bulk signals are broad.

To further support this claim, we performed numerical simulations of the electromagnetic (EM) enhancement of Rh dimers on silicon and aluminum substrates (Supporting Information S1: Figure S15) and their collection efficiency on glass and aluminum films (Supporting Information S1: Figure S16, Note #11 for details). In these simulations, the Rh NCs were modeled as ideal cubes with rounded edges (*r* = 2 nm) and perfect face‐to‐face alignment, as is commonly adopted in the literature [42] (additional simulations considering not perfectly faced NCs and not perfectly cubic NCs are reported in Supporting Information S1: Figure S18 and in Ref. [40]). As shown in Supporting Information S1: Figure S15, the simulations reveal that the Al substrate provides a SERS enhancement factor with log_10_(EF) = 6.1 at 325 nm excitation and log_10_(EF) = 3.7 at 266 nm excitation, which are higher than on Si (log_10_(EF) = 4.6 and 3.4, respectively), due to the formation of a hybrid plasmonic cavity. These quantitative results confirm that the Al substrate plays a dual role: it not only enhances the local electromagnetic field through the mirror effect, but also improves the collection efficiency (Supporting Information S1: Figure S16), together enabling the single‐protein detection sensitivity demonstrated in our experiments.

Given the relatively broad spectral features obtained using the excitation wavelength at 325 nm, we further employed a 266 nm laser (setup presented in Supporting Information S1: Figure S4) to validate the platform's detection performance. Both DNA and some amino‐acids present specific absorbance close to this spectral wavelength, therefore it is interesting to compare the SERS performances at this second excitation wavelength. Under this condition, we tested both streptavidin‐free and streptavidin‐functionalized samples at the low surface density (> 1 μm inter‐dimers distance). The sample was continuously rotated (see Supporting Information S1: Figure S4) during acquisition to ensure that the laser spot did not remain on the same area, thereby significantly reducing cumulative photodamage. This rotation method has been validated in our previous work [39] where we systematically studied the photodegradation dynamics of biomolecules under UV excitation, establishing the necessary conditions for stable signal acquisition. (Here we confirmed that measurements done without rotation produce significant photodegradation in less than 1 min ‐see Supporting Information S1: Figure S10 in Note #8).

The final spectrum (Figure 3) represents the average of 12 acquisitions with an integration time of 5 min each, for a total integration time of 60 min (taking into consideration the rotating sample holder, this spectra represents the average from different Rh dimers). The black spectrum represents the control sample (“streptavidin‐free”) whereas the red spectrum corresponds to the streptavidin‐functionalized sample. The difference between the two spectra is reported in blue, to better enhance the streptavidin spectral features. Results show that a distinct and prominent characteristic peak at approximately 1616 cm^−1^ is clearly observed in the functionalized sample, which corresponds to the peak around 1622 cm^−1^ in Table 1 assigned to Tryptophan Y8b/W1 (to note Tryptophan is the aromatic amino‐acid with the strongest absorbance at 266 nm). At the same time, small peaks at 1050, 1190 and 1340 cm^−1^ can be appreciated in the spectrum. These features can be assigned respectively to W1/NO_3_, Y7A/Y9A/W10 and W7 streptavidin spectral features, recognized in the literature through UV Raman spectroscopy[59]. It provides further confirmation of specific protein detection under optimized UV excitation. To note, it is not trivial to directly compare the SERS enhancements between the measurements performed at 325 and 266 nm. This is because the experimental conditions were radically different. According to the SERS peaks' intensity, it could be possible to speculate that the measurements performed at 325 nm were possible due to a higher enhancement (in agreement with the absorbance properties of the Rh nanostructures), but these signals' intensities are also related to the efficiency of the UV optics and spectrometers, different in the two different experiments.

**FIGURE 3:**
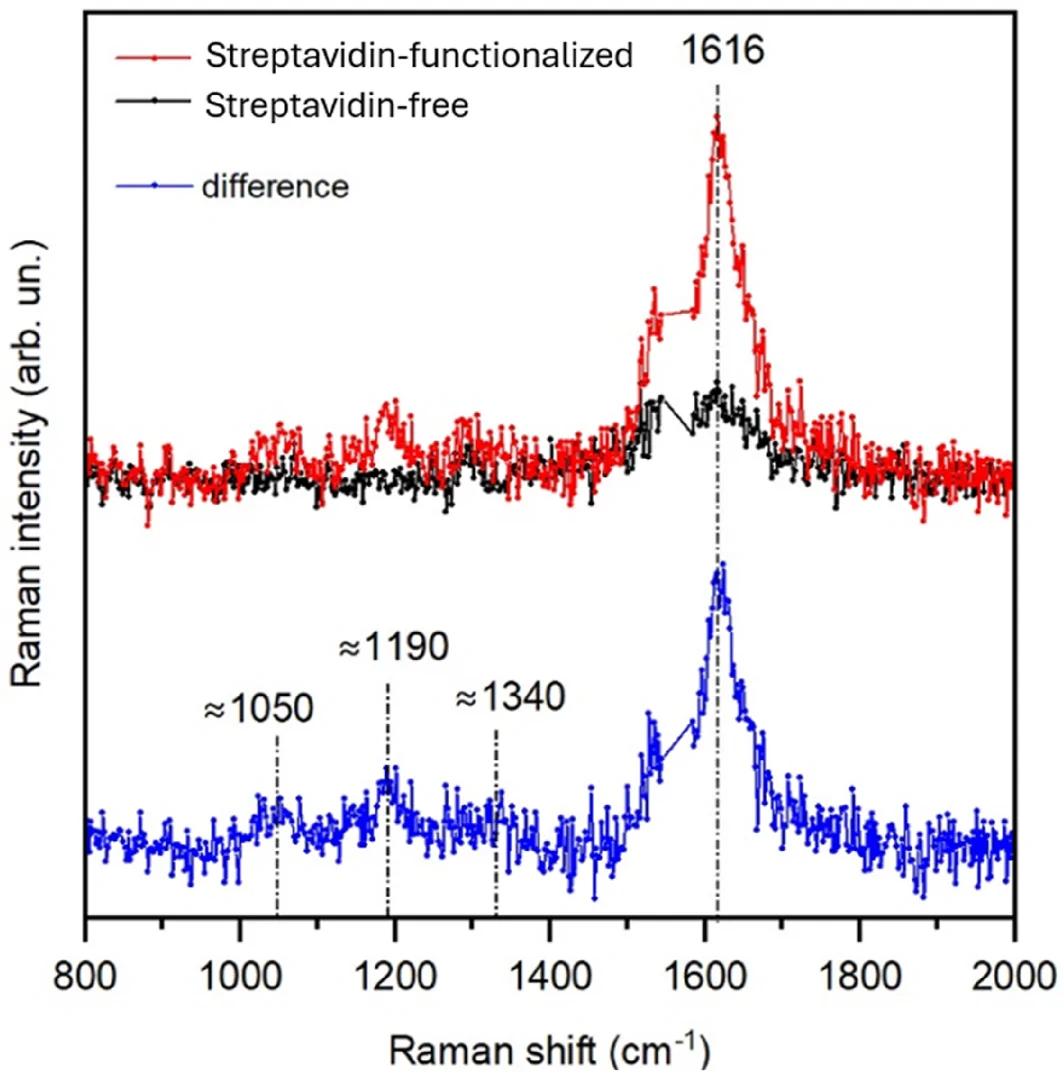
Baseline‐subtracted UV‐SERS spectra under 266 nm excitation. The red spectrum corresponds to the streptavidin‐functionalized sample, the black spectrum to the streptavidin‐free control, and the blue spectrum to their difference. Note the distinct peak at around 1616 cm^−1^, characteristic of streptavidin, which appears exclusively in the functionalized sample.

### 2.3 Validating Polarization‐Controlled UV‐SERS of Streptavidin

The deterministic control over the local electromagnetic field via nanoantenna orientation is a cornerstone of plasmonics, as the excitation of specific plasmon modes depends on the alignment between the incident field and the nanostructure's principal axes [42, 43, 44, 45]. To experimentally validate this principle in our biofabricated system and quantify its impact on single‐molecule detection, we performed comprehensive polarization‐resolved UV‐SERS measurements using both 325 and 266 nm excitation wavelengths (Figure 4). To note, in order to perform this analysis, samples with low spatial density of Rh‐NCs (i.e., << 1 nanostructure per μm^2^) were used. In fact, although the biofabrication ensures the positioning of the dimers on the DNA template, the different structures are then randomly deposited on the Al‐coated silicon substrate; therefore, only analyzing single structures (and the corresponding single protein) it could be possible to obtain a valid polarization‐dependent spectrum.

**FIGURE 4:**
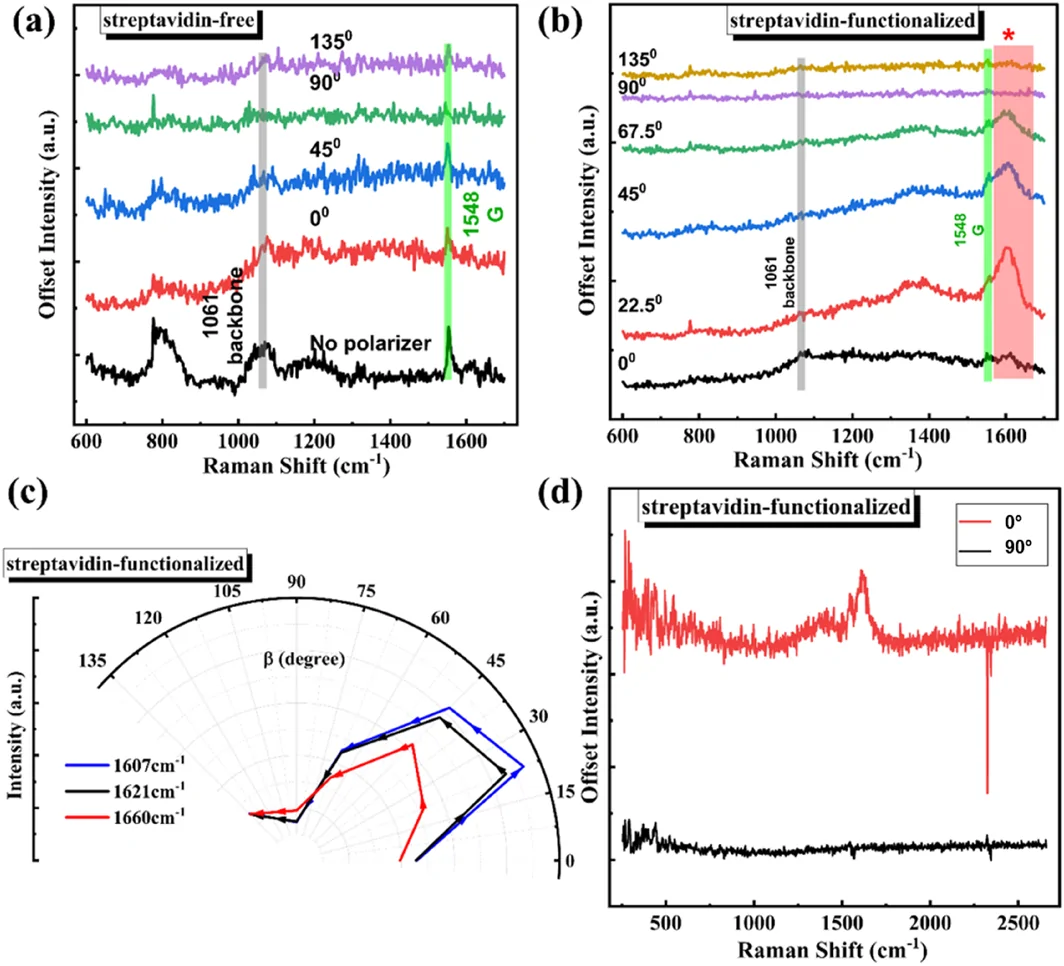
Polarized UV‐SERS spectra of single streptavidin on DNA Origami under 325 and 266 nm excitation. (a–c) 325 nm excitation: (a) UV‐SERS spectra of the streptavidin‐free control sample without a polarizer (black) and with the polarizer rotated from 0° to 135° (color gradient from red to purple). (b) Corresponding SERS spectra of the streptavidin‐functionalized sample at different polarizer angles. (c) Integrated peak intensities at 1607, 1621, and 1660 cm^−1^ (blue, black, and red symbols, respectively) extracted from the spectra in (b). (d) 266 nm excitation: UV‐SERS spectra of the streptavidin‐functionalized sample under 0° and 90° polarized excitation, respectively. Polarization‐dependent spectra were acquired under low laser power with minimal integration time to avoid photodegradation.

As shown in Figure 4a, we initially characterized the spectra of a streptavidin‐free sample as control. We found that the intensity of the peak associated to the DNA structure is reduced by adding a polarizer, with the spectral features only slightly changed with the introduction of the polarizer at different angles, and the intensity of the peak depending on the polarizer orientation. Considering then the streptavidin‐functionalized sample, it is possible to observe how the UV‐SERS intensity (Figure 4b) exhibits a strong and systematic modulation as the polarizer is rotated. For reference, the spectrum acquired without any polarizer is provided in Supporting Information S1: Figure S14 (Note #10), which shows the highest baseline intensity before polarization‐dependent attenuation. Figure 4c clearly presents the quantitative analysis of the integrated intensities for three diagnostic peaks (1607, 1621, and 1660 cm^−1^) extracted from the polarized spectra in Figure 4b. The pronounced intensity modulation with polarization rotation angle reveals a clear dipolar plasmonic response. The maximum intensity for all peaks occurred when the polarizer was rotated by 22.5° from the initial reference (0°) position, whereas the minimum was observed near the orthogonal orientation of 112.5°, indicating that the incident electric field was aligned parallel to the long axis of the NC dimer at this angle. This dependence directly correlates the external optical field with the excitation efficiency of the dimer's fundamental mode, as predicted by simulations in literature [42].

Additional polarization measurements were performed using the excitation wavelength at 266 nm. These second set of measurements confirmed the orientation‐dependent enhancement (Figure 4d). The spectrum acquired without a polarizer in Figure 3 showed an intermediate intensity, representing an incoherent average, whereas the significant modulation obtained with the adjunct of the polarized in Figure 4d directly shows the anisotropy of the plasmonic hotspot engineered by the well‐defined dimer geometry. Collectively, these data provide the first experimental validation of polarization‐selective UV‐SERS in a deterministically assembled DNA‐origami nanoantenna. We demonstrate that the SERS intensity of a single protein is actively modulated by the incident polarization, with maximum enhancement achieved at alignment with the dimer's principal axis under 266 nm excitation. This establishes our platform as providing not only single‐molecule sensitivity but also directional control over the enhancement, a critical advancement for tailored nanophotonic sensing.

## 3 Discussion

Our work establishes a programmable platform that merges DNA nanotechnology (biofabrication) with UV plasmonics to achieve single‐protein detection via label free Raman spectroscopy. We moved beyond conventional gold‐based systems by integrating Rh NCs, whose UV resonances are crucial for exciting biomolecular electronic transitions, into a geometrically precise DNA origami scaffold. This assembly strategy, a decisive step beyond earlier stochastic methods, reliably creates “hot spots” while enabling the deterministic placement of a target molecule.

The observation of characteristic Raman bands from a single streptavidin protein, such as tryptophan and amide vibrations, confirms the platform's specificity and single‐molecule sensitivity in the UV range. More importantly, we provide the first experimental demonstration of polarization‐dependent control in such a DNA‐assembled UV nanoantenna. The modulation of signal intensity with incident polarization—and the clear maximum at a specific angle—directly validates the coupling between the external optical field and the engineered plasmonic mode of the dimer, transforming the structure from a passive amplifier into an active optical element.

These capabilities are fundamentally enabled by a synergistic plasmonic cavity. Our micropatterned control experiments offer visual proof that the aluminum film substrate is indispensable, actively contributing to the enhancement and collection efficiency rather than serving as a passive support. This synergy among material (Rh), geometry (dimer), and substrate (Al) forms the core of our platform's performance.

Looking forward, the inherent programmability of DNA origami provides a clear path for advancement. The scaffold can be redesigned to tune the nanoantenna's spectral response or to present multiple probes for multiplexed detection. The proven polarization control further suggests promising routes toward developing chiral plasmonic sensors. Although challenges remain in areas like large‐scale uniformity and absolute enhancement factor quantification, this work extends the reach of DNA‐assembled plasmonics into the UV regime. It lays a versatile foundation for applications demanding label‐free, single‐molecule spectral fingerprinting with built‐in optical tunability, from fundamental bio‐spectroscopy to next‐generation sensing. Although the present study focuses on Al as the supporting substrate, future work could explore other UV‐plasmonic materials such as Rh thin film to further optimize the hybrid cavity effect and extend the wavelength tunability of the platform.

## 4 Methods

### 4.1 Rhodium Nanoparticles Synthesis

Rhodium nanocubes were synthesized using a seed‐mediated polyol method following Zhang et al. [41]. Potassium bromide and polyvinylpyrrolidone were used as shape‐directing and stabilizing agents, with ethylene glycol acting as solvent and reducing agent. Seed particles were first prepared at 160°C, followed by a controlled growth step via injection of a rhodium precursor into the seed solution. The reaction was maintained at 160°C and then cooled to room temperature, yielding rhodium nanocubes with average edge lengths of approximately 19 and 24 nm. After synthesis, the nanocubes were washed and redispersed in deionized water.

### 4.2 Rhodium Nanocubes DNA Functionalization

After synthesis, rhodium particles were coated with PVP, which were to be removed before DNA functionalization. All procedures, including PVP removal, DNA functionalization, and purification by gel electrophoresis, were performed as described in Corduri et al. [39].

Briefly, PVP was removed by centrifugation and treatment with NaBH_4_ in the presence of Pluronic F‐127. The cleaned rhodium cubes were then mixed with thiolated DNA, and a freeze–thaw method was used to facilitate binding. Finally, purification by gel electrophoresis was employed to remove excess of DNA.

### 4.3 Assembly of DNA Origami “Boat”

DNA origami structures were assembled from a 7249‐nt M13mp18 scaffold using 243 staple strands in 1 × TAE buffer supplemented with 12 mM MgCl_2_ (pH 8), following the protocol described in DNA‐Origami‐Assembled Rhodium Nanoantennas for Deep‐UV Label‐Free Single‐Protein Detection (Corduri et al.). Staples were added in 10‐fold excess over the scaffold (100‐fold for functional staples). The mixture was thermally annealed from 70°C to 25°C. Purification was performed either by agarose gel electrophoresis or by Amicon ultrafiltration. Streptavidin was subsequently added in 20‐fold excess, incubated at room temperature, and the final constructs were purified by agarose gel electrophoresis and stored at 4°C.

### 4.4 Rhodium Dimers Assembly

Rhodium nanocube dimers were assembled by incubating T18‐functionalized rhodium nanocubes with DNA origami at a 20:1 ratio in 1 × TAE buffer containing 12 mM MgCl_2_ and 600 mM NaCl, following the procedure described in Corduri et al., “DNA‐Origami‐Assembled Rhodium Nanoantennas for Deep‐UV Label‐Free Single‐Protein Detection.” The mixture was incubated overnight at room temperature, and the resulting dimers were purified by 1% agarose gel electrophoresis. The corresponding band was extracted and used without further purification.

### 4.5 Deposition Onto Si/Al Substates

To prepare the substrates for the Rh NCs deposition, we used 1 × 1 cm^2^ double‐polished Si wafer (500 μm thick) pieces. After proper cleaning (successive washing in acetone, isopropanol and water), the Si surface was coated with 20 nm of Al evaporated by means of e‐beam in high vacuum. To facilitate the comparison of the SERS performance on Al and on Si, patterned substrates were prepared via simple optical lithography creating small circles of Al over the Si surface (see Figure 1f). Rh NCs dimers (160 pM) were deposited onto aluminum coated (or on Si for control) substrates by incubation for 30 min at room temperature. After deposition, the substrates were gently rinsed with milli‐Q water to remove unbound structures and used for subsequent measurements.

### 4.6 TEM Measurements

For TEM imaging, 5 μL of the solution were dropped onto EM‐Tec Formvar Carbon support film on copper 300 square mesh. After 2 min, the solution was removed with a paper filter, and the sample was stained using 2% Uranyl Acetate for 40 s followed by a quick water rinsing. The measurements were then carried out using a Tecnai Spirit with an accelerating voltage of 120 kV.

### 4.7 Optical Microscopy and Spectroscopy

SERS measurements at 325 nm were performed using a HORIBA LabRAM HR Evolution Raman spectrometer (Horiba Jobin Yvon, Kyoto, Japan) with a 40x UV objective integrated Raman system coupled to a confocal microscope with an 800 mm spectrograph and two switchable gratings. The UV‐SERS spectra were acquired using a 325 nm laser with 500‐hole value. Figure 2a,b were obtained using a 1800gr/mm grating to scan a 70 μm^2^ area, in steps of 14 μm and a total of 36 points. Each point collected with a 270 s exposure time, and a single accumulation at 10% laser power attenuation to minimize photodegradation. Supporting Information S1: Figure S7 was obtained using the same parameter scanning area [−50, 50] μm^2^ with 5 μm step size and a total of 225 points.

UV Raman measurements were performed using a 266 nm excitation wavelength. Diffused light was collected in backscattering configuration by means of a 2‐inch plano‐convex lens with a focal length of 10 cm. The beam power was set to 0.2 mW to minimize sample photodegradation as much as possible. A Czerny–Turner spectrometer (Andor 750), with f/9.4 and a focal length of 750 mm, coupled with a back‐illuminated, Peltier‐cooled CCD (Andor Idus), was used to obtain the final Raman spectra. The spectral resolution was set to 20 cm^−1^ to maximize the count rate. During some measurements, the sample was continually rotated, keeping the focus position on the sample surface, to avoid photodegradation. The final spectra were obtained as the average of 12 spectra of 5 min each. For the polarization measurements, a half‐lambda waveplate was used to set the polarization of the incident light with respect to the sample orientation. Two polarizations were used, namely VV, with the electric field parallel to the sample edge, and V45, with the electric field rotated by 45° with respect to the sample edge. In this case the sample was not rotated and final spectrum were obtained as the average between 5 spectra of 1 min each, collected in different points.

### 4.8 Numerical Simulations

Electromagnetic field maps simulations were performed using the finite element method (FEM) in CST Studio Suite. No adaptive mesh was used. The particles were modeled as cubes with rounded edges of *r* = 2 nm. The permittivity of water was set to 1.77, and the dielectric function of rhodium was taken from “Handbook of Optical Constants of Solids” by Edward D. Palik. The excitation light was linearly polarized either along or perpendicular to the dimer axis.

## Author Contributions

**Yanqiu Zou:** investigation, writing – original draft, methodology, writing – review and editing, data curation. **Nicco Corduri:** investigation, methodology. **Francesco D'Amico:** investigation, methodology. **Karol Kołątaj:** supervision, data curation, methodology. **Huaizhou Jin:** investigation, methodology, writing – review and editing. **Zhenrong Zheng:** funding acquisition, supervision. **Yifan Yu:** methodology, investigation. **Jie Liu:** methodology, investigation. **Shukun Weng:** investigation. **Ali Douaki:** investigation. **Roman Krahne:** resources. **Jerome Wenger:** investigation, methodology. **Shangzhong Jin:** supervision, funding acquisition. **Guillermo Acuna:** supervision, funding acquisition, writing – review and editing, writing – original draft. **Denis Garoli:** investigation, methodology, conceptualization, funding acquisition, writing – original draft, writing – review and editing, supervision, resources, project administration.

## Conflicts of Interest

The authors declare no conflicts of interest.

## Supporting information


Supporting Information S1

## Data Availability

Data are available on request to the authors.
