# Supplementary material for: Self‐Assembled Rhodium Nanoantennas for Single‐Protein UV SERS
Source: Nanophotonics. 2026 May 23;15(10):e70137. doi: 10.1002/nap2.70137 (PMC13642167; doi:10.1002/nap2.70137)
Supplement: Supplementary file 1 [file NAP2-15-e70137-s001.docx]

**Supporting Information**

**Self-assembled Rhodium Nanoantennas for Single-Protein UV SERS**

*Yanqiu Zou^1^, Nicco Corduri^2^, Francesco D’Amico^3^, Karol Kołątaj ^2*^*, *Huaizhou Jin^4^, Zhenrong Zheng^1*^, Yifan Yu^5^, Jie Liu^5^, Shukun Weng^6^, Ali Douaki^6,7^, Roman Krahne^6^, Jerome Wenger^8^, Shangzhong Jin^9^*, *Guillermo Acuna^2*^, Denis Garoli^6,7,9*^*

^1^ State Key Laboratory of Modern Optical Instrumentation, College of Optical Science and Engineering, Zhejiang University, Hangzhou 310027, China

^2^ Department of Physics, University of Fribourg, Chemin du Musée 3, Fribourg CH-1700, Switzerland

^3^ Elettra Sincrotrone Trieste S.C.p.A., S.S. 14 km 163,5 in Area Science Park, 34149,

Basovizza, TS, Italy

^4^ Key Laboratory of Quantum Precision Measurement, College of Physics, Zhejiang University of Technology, Hangzhou, China

^5^ Department of Chemistry, Duke University, Durham, NC 27708, USA

^6^ Istituto Italiano di Tecnologia, via Morego 30, I-16163, Genova, Italy

^7^ Dipartimento di Scienze e Metodi dell’Ingegneria, Università degli Studi di Modena e Reggio Emilia, Via Amendola 2, 42122, Reggio Emilia (Italy)

^8^Aix Marseille Univ, CNRS, Centrale Med, Institut Fresnel, AMUTech, 13013 Marseille, France

^9^ College of Optical and Electronic Technology, China Jiliang University, Hangzhou 310018, China

**Note #1 –TEM characterization of Rh NCs**

**Fig. S1.** TEM micrographs of (a) 19 nm Rh NCs dimers, (b) 24 nm Rh NCs dimers.

**Note #2 – Absorbance spectra of the Rh NCs**

**Fig. S2.** Absorbance spectra of the Rh NCs and Rh dimers. (a) Rh NCs with a size of 24 nm; (b) Rh NCs with a size of 19 nm.

**Note #3 – Raman setup**

**Fig. S3.** Raman spectrometer setup for the measurements at 325 nm.

**Fig. S4.** Details on the Raman spectrometer for the measurements at 266 nm – sample+polarizer holders.

**Note #4 – Origami + Rh NCs configurations**

While detailed modelling of Rh NCs configurations are reported in AdvancedFunctionalMaterials,2026;0:e32006 (<https://doi.org/10.1002/adfm.202532006>), Fig. S5 illustrates the two potential orientations.

**Fig. S5.** Potential configurations of the origami+Rh NCs after the drop cast on the substrate.

**Note #5 – UV-SERS mapping and spectral analysis of Rh dimer hotspots for streptavidin detection**

The UV-SERS mapping was performed over the 1055–1067 cm⁻¹ region to visualize the spatial distribution of the DNA backbone peak at 1061 cm⁻¹, which serves as a positional marker for the DNA origami nanostructures. The maps in Fig. S6(a) and 6(b) were generated from the normalized data presented in Fig. 2a and 2d, respectively. The difference spectrum in Fig. S6 (c) was obtained by subtracting the streptavidin-free average spectrum from the streptavidin-functionalized one. Deconvolution of the difference spectrum Fig. S6(d) was performed using a multi-peak fitting routine with Gaussian line shapes, yielding an excellent fit ($R^{2}=0.99606$). The characteristic Raman peaks correspond to vibrational modes of streptavidin and the DNA origami scaffold, further confirming specific binding.

**Fig. S6.** UV-SERS mapping of the streptavidin-free **(a)** and streptavidin-functionalized **(b)** samples from **Fig. 2a and 2d** integrating over the 1055-1067 cm^-1^ region (associated with 1061cm^-1^ DNA backbone peak), respectively. **(c)** Averaged UV-SERS spectra (±SD) of streptavidin-functionalized (red) and streptavidin-free (black) samples，with difference spectrum (blue) shown below; **(d)** Deconvolution of the difference spectrum (black) into individual Raman components (colored peaks) and cumulative fit (red dashed). Peak positions (cm^-1^) are labeled, and the fit quality is reflected by *R^2^* = 0.99606.

**Note #6 – UV-SERS spectra Comparison of Streptavidin-Functionalized 19 nm and 24 nm Rhodium NCs on DNA Origami at 325nm**

**Fig. S7.** UV-SERS 2D mapping and extracted spectra of low-surface-density (inter-dimers distance >>1μm) streptavidin-functionalized Rh NCs dimers. (a-b) UV‑SERS 2D mapping obtained with an 1800 gr/mm grating for (a) 19 nm and (b) 24 nm Rh NCs dimers. (c-d) Stacked plots of three representative spectra extracted from the corresponding 2D maps in (a) and (b), respectively. Measurement conditions: 325 nm excitation, 10% laser power, 270 s integration time per point, 5 μm step size, 225 points (15 × 15 grid), scanning area [-50,50] μm^2^.

To quantitatively assess the reliability and reproducibility of our UV-SERS platform, we performed 2D mapping and statistical analysis of the SERS signals for both 19 nm and 24 nm Rh NC dimers, following the single-molecule SERS detection criteria established by Kneipp et al^1^. As shown in Fig. S7(a, b), UV-SERS 2D mapping was performed over a scanning area of [-50,50] μm^2^ with a step size of 5 μm, resulting in a total of 225 points (15 × 15 grid) for each sample. The corresponding representative spectra extracted from the maps are displayed in Fig. S7(c, d). For the 24 nm Rh NC dimers (Fig. S7b), a higher density of bright hotspots is observed compared to the 19 nm dimers (Fig. S7a), indicating stronger SERS enhancement. Consistently, the representative spectra from the 24 nm sample (Fig. S7d) exhibit more intense and reproducible spectral features associated with streptavidin, including the characteristic Y8 peak at ~1595.7 cm⁻¹, whereas the 19 nm sample (Fig. S7c) shows weaker and less consistent signals.

**Fig. S8.** Statistical analysis of UV-SERS signals for 19 nm and 24 nm Rh NCs dimers. (a-b) Peak intensities at 1595.7 cm^-1^ extracted from 225 mapping points for streptavidin-free samples with (a) 19nm and (b) 24nm Rh NCs, respectively. The mean value, and the standard deviation (SD) are provided, and the detection threshold for protein signals, defined as three times the standard deviation of this background noise (3σ), is marked with red horizontal line. (c-d) Peak heights of the 1595.7 cm^-1^ for streptavidin-functionalized samples (shown in Fig. S7a-b) with (c) 19 nm (blue) and (d) 24 nm (green) Rh NCs, respectively.

To further quantify these observations, we performed a statistical analysis of the peak intensities at ~1595.7 cm⁻¹ extracted from all 225 mapping points for each sample (Fig. S8). The background noise level (σ) was determined from the streptavidin-free control samples (without protein) for both 19 nm and 24 nm Rh NCs. Following the single-molecule SERS detection criteria established by Kneipp et al^1^. We set the detection threshold for protein signals at three times the standard deviation of this background noise (3σ), as indicated by the red horizontal lines in Fig. S8. For the 19 nm sample, the 3σ threshold was 13.5, while for the 24 nm sample, the 3σ threshold was 23.3.

Importantly, a striking difference lies in the detection yield and signal distribution. For the 19 nm sample (Fig. S8c), a noticeable fraction of points still falls below the 3σ threshold, indicating that not all hotspots successfully captured a protein or generated detectable SERS signals. In contrast, for the 24 nm sample (Fig. S8d), all 225 mapping points exceed the 3σ threshold, demonstrating a 100% detection yield. Furthermore, only the 24 nm dimers produce ultra-high-intensity events (>1000 a.u., up to ~5000 a.u.), which are completely absent in the 19 nm sample (all points below 400 a.u.). These results indicate that while both platforms enable protein detection, the 24 nm Rh NC dimers provide superior electromagnetic field enhancement and more reliable single-protein detection, likely due to more efficient plasmonic coupling and better spectral overlap with the excitation wavelength.

**Note #7 - Substrate‑Specific Control on Micropatterned Substrates at 325 nm**

We tested the system at a low DNA‑origami assembly density (average inter‑dimer spacing >1 μm). The samples were prepared both with and without the target protein, streptavidin. The full set of correlative characterization data—including large‑area SERS maps, spatially resolved intensity profiles of the characteristic DNA band at 1061 cm⁻¹, and high‑resolution AFM topographs—is compiled in Fig. S9.

The results demonstrate a definitive, binary response: characteristic SERS signals (from either the DNA scaffold or the captured protein) were exclusively detected on very few points (due to the low spatial density of the structure) on the aluminum stripes. No measurable signal above the noise background was obtained from the adjacent Si regions. This clear spatial contrast provides direct and visual evidence that the observed UV‑SERS enhancement is an intrinsic property of the Rh NCs dimers that on the Al substrate generate a plasmonic cavity also supported by a larger collection efficiency (see Fig. S11) an effect not supported by the Si substrate.

**Fig. S9.** Substrate‑specific UV‑SERS response on Al/Si micropatterns with 0.5X DNA‑origami assembly density. a) UV‑SERS mapping spectra of the DNA‑origami scaffold without streptavidin. Inset: AFM topography (70 μm × 70 μm) of scanned region, the circle represents the Al coated area. b) Zoomed AFM image (5μm × 5 μm) corresponding to the area in a). c) Corresponding SERS intensity map derived from the DNA phosphate backbone band at 1061 cm⁻¹ in a). d) UV‑SERS mapping spectra of the streptavidin-functionalized sample. e) AFM image (5μm × 5 μm) of streptavidin-functionalized region and f) SERS intensity map at 1061 cm⁻¹ extracted from d).

**Note #8 - Photodegradation tests at 266 nm**

**Fig. S10.** Photodegradation tests at 266 nm excitation wavelength. Measurements done with the sample at a fixed position, centered to a Rh dimer.

**Note #9 - Control samples on Silicon Substrates at 325 nm**

As shown in Fig. S11a, the Si sample serves as a reference. After the deposition of the DNA‑origami‑assembled Rh‑NCs dimer sample (Fig. S11b) it is possible to observe some increased signals. The number of observable peaks is clearly lower with respect to the samples prepared on Al even if it is still possible to distinct a DNA‑related vibrational peak at approximately 1555 cm⁻¹, which is absent in the Si reference. This peak confirms the presence and structural integrity of the DNA scaffold in the assembled nanoantenna.

**Fig. S11** UV-SERS spectra of a) silicon and b) DNA Origami with Rh NCs (streptavidin-free) on silicon substrates. Spectra were collected from randomly selected points under 325 nm excitation (40× NUV objective). For signal optimization, laser filters were set to 10 % for silicon and 50 % for DNA‑origami samples.

To systematically evaluate the contribution of the Rh NCs and the streptavidin functionalization to the observed SERS signals, control SERS mapping experiments were performed under 325 nm excitation (Fig. S12). Two control samples were examined: DNA‑origami with streptavidin without Rh NCs (a), and bare DNA‑origami (without the protein) (b). The corresponding averaged SERS spectra confirm that the SERS enhancement is primarily mediated by the plasmonic activity of the Rh NCs dimers, and that the streptavidin functionalization, in the absence of nanoparticles, does not yield measurable SERS activity under these experimental conditions.

**Fig. S12** UV-SERS mapping of control samples. SERS mapping performed under 325 nm excitation with a 10% laser power filter and 1800 gr/mm grating for the following samples: a) DNA‑origami functionalized with streptavidin and assembled without Rhodium nanoparticles; b) bare DNA‑origami without the protein and the Rh NCs.

To further support the assignment of the SERS peaks observed at the Rh dimer hotspots, we measured the normal Raman spectra of bulk streptavidin (1 mg/mL) powder under the same excitation conditions (325 nm, 10% laser power, 270 s acquisition). As shown in Fig. S13, the bulk spectrum of streptavidin exhibits broad features, which are consistent with the UV-SERS spectra of the streptavidin-functionalized samples.

**Fig. S13** UV Raman spectra of bulk streptavidin on silicon substrates. Mean spectrum of 1mg/ml Streptavidin in 1X PBS solution (black line) with error bar representing standard deviation (n=4). Measurement conditions: 325 nm excitation, 10% laser power filter, 270 s acquisition time, 1800 gr/mm grating.

**Note #10 - UV-SERS spectra without polarizer at 325nm**

**Fig. S14.** UV‑SERS spectra of the streptavidin‑functionalized sample under 325 nm excitation without a polarizer.

Fig. S14 presents the UV‑SERS spectrum of the streptavidin‑functionalized sample acquired without a polarizer. This spectrum serves as a reference and demonstrates that the signal intensity is significantly reduced when a polarizer is introduced and rotated, as shown in the polarization‑dependent measurements in Fig. 4b of the main text.

**Note #11 – Numerical Simulations**

The SERS enhancement factor (EF) is a key metric that quantifies the plasmonic performance of a nanostructure, which represents the factor by which the Raman signal is amplified relative to normal Raman scattering. For SERS EM-enhancement, the EF is proportional to the fourth power of the local electric field enhancement, i.e., EF = |E|⁴/|E₀|⁴, where |E| is the local electric field at the hotspot and |E₀| is the incident field^33^. This |E|⁴ approximation has been rigorously justified by Le Ru and Etchegoin using the optical reciprocity theorem^3^, and is widely used in the literature for estimating SERS enhancement factors from numerical simulations. Higher EF values indicate stronger electromagnetic field confinement and greater signal amplification, which are essential for achieving single-molecule sensitivity^3^.

**Fig. S15** Numerical Simulations – Comparison between the two configurations: Rh NCs on Si (left column) and Rh NCs on Al (right column). Top panels: polarization parallel to the dimer axis (E); bottom panels: polarization perpendicular to the dimer axis (B). The values indicate log₁₀(EF), where EF = |E|⁴/|E₀|⁴. Excitation wavelengths are 266 nm and 325 nm as indicated.

The simulated SERS enhancement factors are presented in Fig. S15 as log₁₀(EF) for Rh dimers on Si and Al substrates under 266 nm and 325 nm excitation. Two polarization configurations are considered: **E**: polarization parallel to the dimer axis (top panels) and **B**: polarization perpendicular to the dimer axis (bottom panels), the corresponding enhancement factors (EF) can be calculated as EF = 10^(log₁₀(EF)^. The simulations reveal that the Al substrate provides significantly higher enhancement compared to Si, particularly at 325 nm excitation with parallel polarization (log₁₀(EF) = 6.1 vs 4.6 on Si), due to the formation of a hybrid plasmonic cavity between the Rh dimers and the Al mirror.

**Fig. S16** Illustration of the collection efficiency with and without the Al mirror below the Rh NCs. (a) Rh NCs dimer on Al film; (b) Rh NCs dimer on glass.

**Fig. S17.** SEM micrograph of a large number of 24nm Rh cubes post PVP removal and DNA functionalization (no DNA origami). Inset: Average measurement of the concave shape (obtained from 20 different cubes), gap between the red line and the cube’s central faced: 2nm.

**Fig. S18.** FEM simulations of Rh cubes on Al. Comparison between full cube (E.M. norm 3.9 V/m) and concave cube (E.M. norm 2.9 V/m). Electric field norm at 266 nm, light polarized parallel to the dimer. 24 nm Rh nanocube dimers separated by a 10 nm gap, on aluminum Al surface. The electric field norm is evaluated at the position of the streptavidin molecule (designated by the black dot).

**References**

(1)	Kneipp, K.; Wang, Y.; Kneipp, H.; Perelman, L. T.; Itzkan, I.; Dasari, R. R.; Feld, M. S. Single Molecule Detection Using Surface-Enhanced Raman Scattering (SERS). *Phys. Rev. Lett.* **1997**, *78* (9), 1667–1670. https://doi.org/10.1103/PhysRevLett.78.1667.

(2)	Le Ru, E. C.; Blackie, E.; Meyer, M.; Etchegoin, P. G. Surface Enhanced Raman Scattering Enhancement Factors: A Comprehensive Study. *J. Phys. Chem. C* **2007**, *111* (37), 13794–13803. https://doi.org/10.1021/jp0687908.

(3)	Le Ru, E. C.; Etchegoin, P. G. Rigorous Justification of the |E|4 Enhancement Factor in Surface Enhanced Raman Spectroscopy. *Chem. Phys. Lett.* **2006**, *423* (1–3), 63–66. https://doi.org/10.1016/j.cplett.2006.03.042.

(4)	Le Ru, E. C.; Etchegoin, P. G.; Meyer, M. Enhancement Factor Distribution around a Single Surface-Enhanced Raman Scattering Hot Spot and Its Relation to Single Molecule Detection. *J. Chem. Phys.* **2006**, *125* (20), 204701. https://doi.org/10.1063/1.2390694.
